# Removing cartilage in the “unworn” side increases the accuracy in restoring the distal joint line in kinematic alignment total knee arthroplasty

**DOI:** 10.1002/jeo2.70618

**Published:** 2026-04-23

**Authors:** Giorgio Cacciola, Daniele Vezza, Francesco Bosco, Francesco Carturan, Alessandro Massè, Luigi Sabatini

**Affiliations:** ^1^ Department of Orthopedics and Traumatology University of Turin Turin Italy; ^2^ Deparment of Robotic and Mini‐Invasive Orthopedics Surgery Humanitas “Gradenigo” Hospital Turin Italy; ^3^ Department of Precision Medicine in Medical, Surgical and Critical Care (Me.Pre.C.C.) University of Palermo Palermo Italy; ^4^ Department of Orthopaedics and Traumatology, G.F. Ingrassia Hospital Unit, ASP 6 Palermo Italy

**Keywords:** cartilage, joint line, kinematic alignment, LDFA, unworn

## Abstract

**Purpose:**

In kinematically aligned total knee arthroplasty (KA‐TKA), the restoration of the native joint line is critical to achieving optimal functional outcomes. The conventional assumption of a uniform 2 mm cartilage thickness may lead to errors in distal femoral resections. This study aimed to evaluate whether systematic cartilage removal from the unworn femoral condyle improves the accuracy and consistency of distal joint‐line restoration.

**Methods:**

A retrospective analysis was conducted on prospectively collected data from 374 patients who underwent primary KA‐TKA between March 2023 and March 2025 at a single institution. Patients were divided into two groups: Group A (*n* = 187), where cartilage on the unworn femoral condyle was preserved, and Group B (*n* = 187), where cartilage was removed before distal resection. All procedures used the same surgical technique and implant model. The lateral distal femoral angle (LDFA) was measured preoperatively and postoperatively on full‐length weight‐bearing radiographs. The LDFA restoration error was defined as the difference between postoperative and preoperative LDFA values. Group comparisons were performed using the Wilcoxon–Mann–Whitney *U* test and Levene's test for equality of variances.

**Results:**

Group B (cartilage removed) showed a significantly lower LDFA restoration error (–0.12° ± 0.94°) compared to Group A (–0.35° ± 1.88°) (*p* < 0.001). Levene's test confirmed a significantly reduced dispersion of LDFA error in Group B (*p* = 0.027). A greater proportion of Group B patients achieved LDFA restoration within ±0.5° (52.9% vs. 38.5%) and ±1° (85.0% vs. 71.7%) of the target value (*p* < 0.001 for both).

**Conclusions:**

Removing cartilage from the unworn femoral condyle significantly enhances the precision and consistency of distal joint line restoration in KA‐TKA. This technical refinement may reduce alignment variability and improve reproducibility by addressing interindividual differences in cartilage thickness, which are often underestimated in standard practice.

**Level of Evidence:**

Level III, retrospective comparative study.

## Graphical abstract

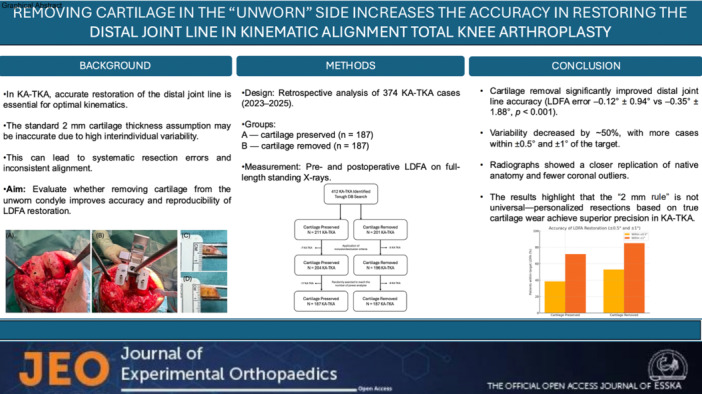

**Abbreviations**
CT: computed tomography
IQR: interquartile range
IRB: Institutional Review Board
KA‐TKA: kinematically aligned total knee arthroplasty
LDFA: lateral distal femoral angle
MRI: magnetic resonance imaging
PROMs: Patient‐Reported Outcome Measures
TKA: total knee arthroplasty

## INTRODUCTION

Over the past decade, numerous innovations have been introduced into clinical practice to address this gap in patient satisfaction [1, 2, 3, 4, 5]. One of the most extensively explored approaches is the restoration of the knee's native kinematics [13, 19]. Given the complexity of native knee motion, it is believed that approximating its restoration as closely as possible may lead to improved functional outcomes [24]. This has led to an increase in the use of medial pivoting implants in recent years, as well as to the introduction of the kinematic alignment (KA) philosophy [11, 20, 25].

The philosophy behind the development of KA stems from biomechanical studies by Freeman, Hollister, Coughlin, Eckhoff and Iranpour, who theorised the three kinematic axes of the knee that govern the reciprocal movements between the tibia, femur and patella [6, 7, 14]. These three axes maintain an orthogonal relationship throughout the entire range of motion; therefore, the goal of KA is to perform bone cuts and position the components in a way that preserves the integrity of these three axes [6, 7, 14].

The surgical technique described by Howell is based on anatomical resurfacing of the distal and posterior femoral condyles, removing bone and cartilage to a depth corresponding to the thickness of the femoral component to be implanted [16]. Traditionally, it assumes a uniform cartilage thickness of 2 mm; thus, resections on the worn side are reduced by 2 mm, while on intact cartilage, the full 2 mm is considered [12, 16].

However, recent studies challenge this assumption, showing considerable interindividual variability [9, 10, 15]. Klasan et al. [15], using intraoperative data from the Mako Total Knee SmartRobotics™, found that in 42 patients, cartilage thickness on the distal unworn femoral condyle exceeded 2.5 mm in 16.7% and was below 1.5 mm in 35% of cases. Similarly, Giurazza et al. [9] measured cartilage thickness intraoperatively using electrocautery, reporting a median value of 2.5 mm.

In a systematic review of 27 magnetic resonance imaging (MRI)‐based studies, the same group reported mean cartilage thicknesses of 2.05 ± 0.62 mm on the distal medial and 1.95 ± 0.4 mm on the distal lateral femoral condyle across 8170 MRIs [10]. Notably, they highlighted substantial variability, with values reaching up to 4.4 ± 1.4 mm, underscoring the importance of individualised anatomical assessment.

Despite the centrality of joint line restoration in kinematically aligned total knee arthroplasty (KA‐TKA), most techniques still assume a uniform approximately 2 mm distal cartilage on the unworn condyle. Evidence shows substantial inter‐individual variability, which may propagate systematic errors in distal resections and increase alignment dispersion. Addressing this gap could improve reproducibility and reduce outliers in coronal alignment.

It was hypothesised that removal of cartilage from the unworn femoral condyle would (i) reduce the lateral distal femoral angle (LDFA) restoration error and (ii) decrease its dispersion compared with cartilage preservation. The aim of this study was to determine whether this technical modification improves the accuracy and consistency of distal joint line restoration in KA‐TKA.

## MATERIALS AND METHODS

### Study design

After institutional review board approval was obtained (IRB 123/2024 AOU Città della salute e Della Scienza, Turin, Italy), prospectively collected data from patients who underwent primary KA‐TKA for severe knee osteoarthritis (KL grade III or IV) between September 2023 and March 2025 were retrospectively analysed. All knees were included in the study and managed with the same surgical technique (unrestricted KA), regardless of the deformity type (valgus or varus) or severity. Our internal database prospectively collects demographic, clinical, surgical, and radiographic data from all patients undergoing prosthetic surgery. The database is anonymized so that patients cannot be identified from the collected data. During the study period, 412 patients underwent KA‐TKA. Patients were included in the study if preoperative and postoperative full‐length weight‐bearing radiographs of the lower limbs were available and of sufficient quality to allow accurate measurement of radiographic parameters related to lower limb alignment. Patients were excluded if intraoperative issues arose concerning the thickness of the distal femoral resections (i.e., resections less than 6 mm on the worn condyle or less than 8 mm on the unworn condyle) or in cases of previous lower‐limb fractures or surgical procedures, as osteotomies were performed. After applying inclusion and exclusion criteria, 374 patients were finally included in the analysis.

### Surgical technique

All patients underwent the same surgical technique, known as calipered, unrestricted manual KA, with the implantation of the identical prosthetic component (GMK SpheriKA, Medacta International). Before surgery, demographic, clinical and radiographic data were collected for each patient. During the surgical procedure, the thickness of the distal and posterior femoral resections, tibial resection and implant sizes were documented. The posterior cruciate ligament (PCL) was preserved in 198 cases, accounting for 52.9% of the procedures. PCL was routinely sacrificed until March 2024, then was routinely preserved. The patella was never resurfaced. A 10 mm liner was implanted in 348 cases (88.3%), an 11 mm liner in 15 cases (3.8%), a 12 mm liner in 23 cases (5.8%) and a 14 mm liner in eight cases (2.0%)

**Figure 1:**
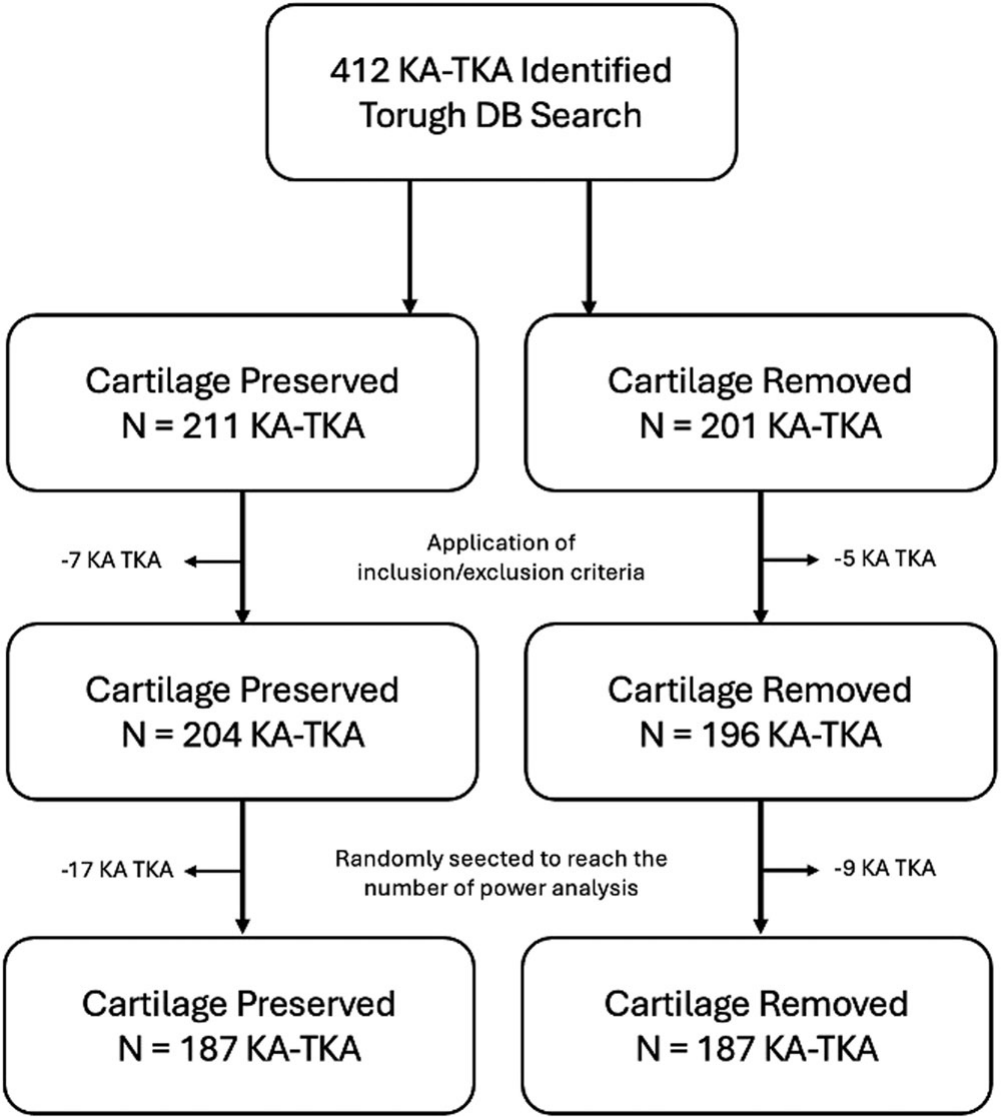
A total of 412 KA‐TKA were identified through the institutional database search. Patients were divided into two groups based on the surgical approach to the cartilage of the unworn femoral condyle: 211 in the cartilage‐preserved group and 201 in the cartilage‐removed group. After applying inclusion and exclusion criteria, 204 KA‐TKA remained in the cartilage‐preserved group and 196 in the cartilage‐removed group. To meet the sample size required by the a priori power analysis, 187 patients were randomly selected from each group for the final analysis. DB, databases; KA‐TKA, kinematic alignment‐total knee arthroplasty; N, number of evaluation cases.

Patients were categorised into two groups based on whether the maintenance cartilage was retained or removed from the unworn condyle. Until May 2024, the cartilage on the unworn condyle was preserved in all cases. The distal femoral cut was performed using the “worn‐unworn” cutting blocks from the MIKA instrumentation set (Medacta International), allowing for a 6‐mm resection on the worn condyle and an 8‐mm resection on the unworn condyle (Figure 1).

**Figure 2:**
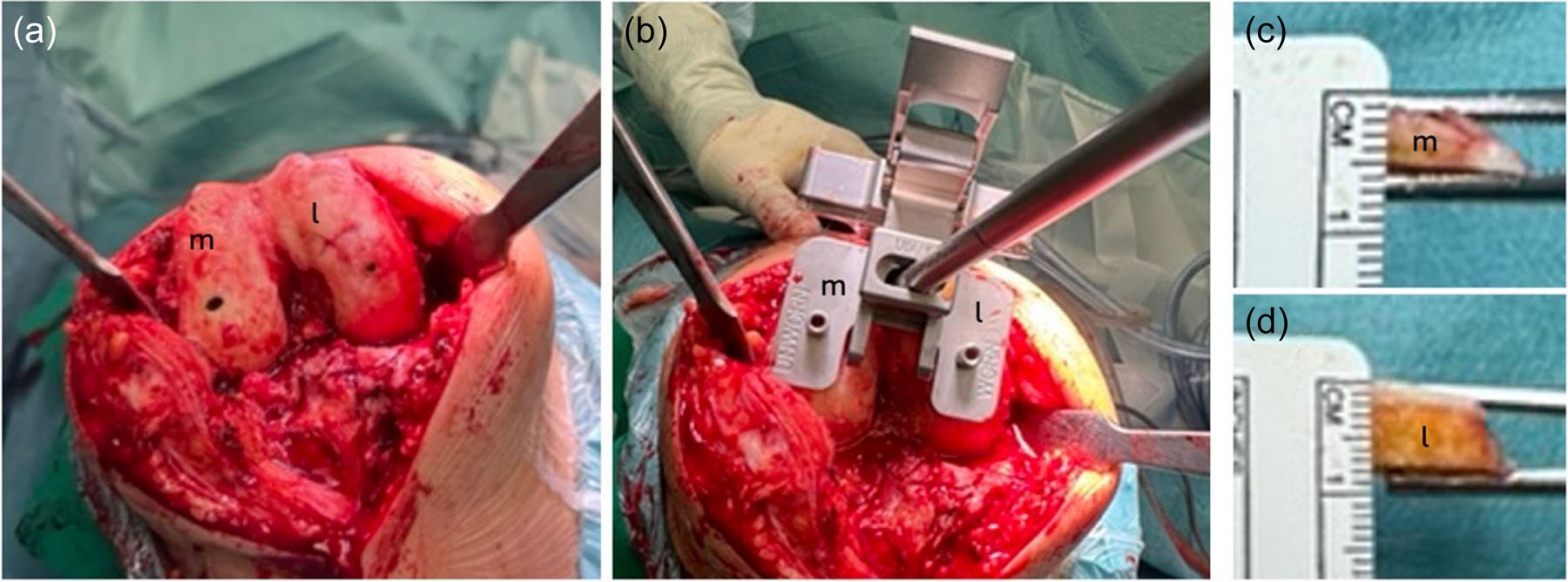
Standard distal femoral resection technique assuming a uniform cartilage thickness of 2 mm: (a) Assessment of distal femoral cartilage: absence of cartilage on the medial condyle and preserved cartilage on the lateral condyle. (b) Distal femoral resection using the “medial/worn” and “lateral/unworn” cutting guides, resulting in a medial cut of 6 mm of bone plus 1 mm for saw blade thickness, and a lateral cut of 6 mm of bone plus 2 mm of cartilage plus 1 mm for saw blade thickness. (c) Verification of the resection on the medial distal femoral condyle. (d) Verification of the resection on the lateral distal femoral condyle. l, lateral side; M, medial side.

From June 2024 onward, cartilage from the unworn compartment was removed using a scalpel, enabling a uniform 6 mm bony resection on both the medial and lateral condyles (Figure 2). Complete removal of the cartilage allowed bone resections to be performed directly on the subchondral surface, theoretically eliminating the asymmetry caused by differential cartilage wear. Bone resection thickness was subsequently adjusted to match the total amount of tissue removed to the thickness of the femoral component, ensuring restoration of the distal joint line height.

### Radiographic analysis

A long‐leg weight‐bearing radiograph of the lower limb was acquired preoperatively, and a follow‐up radiograph was obtained at the first clinical examination two months after surgery. One of the authors (G.C.) reviewed all radiographs and collected preoperative and postoperative LDFA measurements without knowledge of group allocation (Group A or B).

LDFA was defined as the lateral angle between the femoral anatomical axis and the distal femoral joint line on full‐length standing radiographs. The femoral axis was determined by connecting two mid‐diaphyseal points separated by at least 10 cm. At the same time, the distal joint line was traced as a tangent to the most distal aspects of the femoral condyles. According to Paley's definition, a physiological LDFA ranges between 87° and 88° in normal alignment [18]. The first author (G.C.) calculated the LDFA restoration error by subtracting the preoperative LDFA value from the postoperative LDFA value. A negative value indicated a more valgus orientation of the distal femur postoperatively. DICOM files were imported into the Horos DICOM viewer (version 3.3.6; Mac OS), and the measurement accuracy was 0.1°. To assess reliability, two blinded observers independently measured LDFA in a random subsample of 60 knees and repeated the measurements 2 weeks later. Intra‐ and inter‐rater reliability were calculated using the intraclass correlation coefficient (ICC, two‐way random‐effects model), and the standard error of measurement (SEM) was computed as SD × √(1–ICC). The analysis demonstrated excellent repeatability, with intra‐rater ICC = 0.96, inter‐rater ICC = 0.95, and SEM = 0.18°.

Preoperative alignment phenotype was determined using the Coronal Plane Alignment of the Knee (CPAK) classification, as described by MacDessi et al. [17]. The CPAK system integrates the arithmetic hip–knee–ankle (HKA) angle and the joint line obliquity (JLO) to categorise coronal knee phenotypes into six alignment types. Classification was performed on preoperative long‐leg radiographs by two independent observers blinded to surgical group assignment. Discrepancies were resolved by consensus discussion. The CPAK distribution was subsequently compared between groups to verify baseline equivalence.

### Statistical analysis

A priori power analysis was conducted to determine the required sample size using the Wilcoxon–Mann–Whitney test, considering potential non‐normal distributions and unequal variances between groups. Based on preliminary institutional data, a mean difference of 0.25° in distal femoral resection error was expected. Following established guidelines for nonparametric power estimation, a total of 374 subjects (187 per group) were required to achieve 90% statistical power with a two‐tailed significance level of 5%. Postoperative LDFA values were collected for each patient and compared to the corresponding preoperative measurements. The LDFA restoration error was calculated as the difference between postoperative and preoperative values (Postoperative LDFA − Preoperative LDFA). A negative value indicates a more valgus‐oriented femur postoperatively. The Wilcoxon–Mann–Whitney *U* test was used to assess differences in restoration accuracy between groups, with a significance threshold set at *p* < 0.05. To evaluate differences in variability between groups, Levene's test for equality of variances was applied to the LDFA restoration error data. The test was performed using median‐centred values to improve robustness against non‐Gaussian distributions. A *p*‐value < 0.05 was considered indicative of a statistically significant difference in dispersion between groups. All statistical analyses were conducted using GraphPad Prism 7.0 (GraphPad Software, La Jolla, CA, USA).

## RESULTS

**Figure 3:**
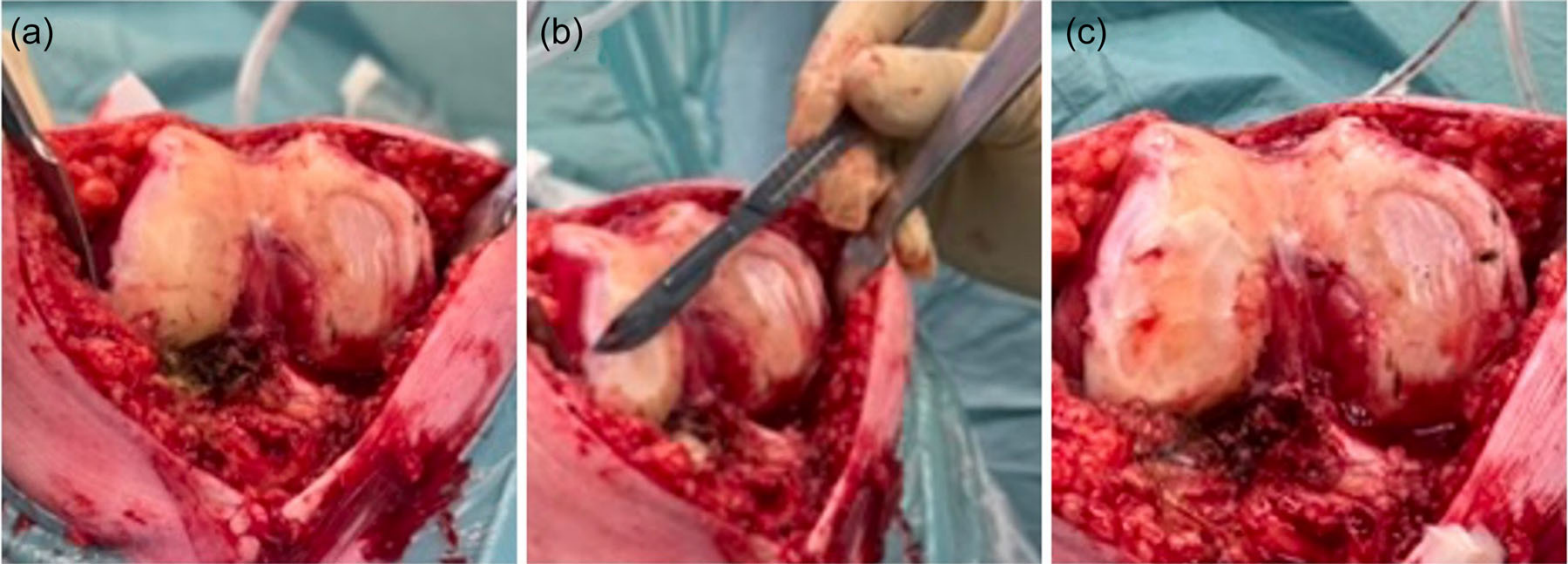
Preparation of the distal femoral condyles for the use of the “worn/worn” cutting guide: (a) Evaluation of cartilage thickness on the medial and lateral distal femoral condyles using a calipered pin. (b) Removal of cartilage from the “unworn” condyle using a scalpel. (c) With both condylar surfaces now free of cartilage, the “worn/worn” distal femoral cutting guide can be positioned. The thickness of the bone resection should be adjusted according to the previously measured cartilage thickness to match the exact thickness of the prosthetic component.

A total of 412 patients who underwent KA‐TKA during the study period were available for inclusion (Figure 3). After applying the eligibility criteria, 374 patients were randomly selected for analysis and evenly divided into two groups: 187 patients in Group A (cartilage on the unworn femoral condyle preserved) and 187 patients in Group B (cartilage on the unworn femoral condyle removed). The two groups were comparable at baseline with respect to demographic characteristics and preoperative alignment (Table 1). Patients in the two groups did not differ for the preoperative CPAK classification (Table 2).

**Table 1:** This table shows the baseline demographic characteristics of the two groups.

Variable	Group A (*N* = 187)	Group B (*N* = 187)	*p* value
Age (years), mean ± SD	71.2 ± 6.9	70.8 ± 7.1	0.52
Sex (M/F), mean ± SD	72/115	68/119	0.64
BMI (kg/m²), mean ± SD	28.3 ± 3.7	28.1 ± 3.9	0.71
Operated side (R/L), N/N	102/85	98/89	0.67
Preoperative LDFA (°), mean ± SD	87.3 ± 2.1	87.5 ± 2.0	0.44
Preoperative HKA (°), mean ± SD	173.3 ± 6.1	172.8 ± 5.8	0.41
– Primary osteoarthritis, *N* (%)	180 (96.3%)	181 (96.8%)	0.79
– Post‐traumatic arthritis, *N* (%)	5 (2.7%)	4 (2.1%)	0.73
– Inflammatory arthritis, *N* (%)	2 (1.1%)	2 (1.1%)	1.00

*Note*: No significant differences were found between groups in terms of age, sex, BMI, operated side or diagnosis.

Abbreviations: %, percentage; °, degree; BMI, body mass index; F, female; L, left; LDFA, lateral distal femoral angle; M, male; N, number of evaluation cases; R, right; SD, standard deviation.

**Table 2:** This table summarises the preoperative distribution of CPAK types between Group A and Group B (*N* = 187 per group).

CPAK classification	Group A (*N* = 187)	Group B (*N* = 187)	*p* value
CPAK I	49 (26.2%)	36 (19.3%)	0.139
CPAK II	75 (40.1%)	63 (33.7%)	0.238
CPAK III	18 (9.6%)	29 (15.5%)	0.119
CPAK IV	10 (5.3%)	18 (9.6%)	0.169
CPAK V	29 (15.5%)	27 (14.4%)	0.885
CPAK VI	6 (3.2%)	14 (7.5%)	0.108

*Note*: In both cohorts, CPAK II was the most prevalent pattern, followed by CPAK I. Group B showed relatively higher proportions of CPAK III, IV and VI compared to Group A, whereas CPAK I and II were more frequent in Group A. Statistical comparison did not reveal significant differences between groups for any CPAK type.

Abbreviation: CPAK, coronal plane alignment of the knee.

The LDFA restoration error, defined as the difference between postoperative and preoperative LDFA values (Postoperative LDFA – Preoperative LDFA), was significantly lower in Group B compared to Group A. In Group A, the mean LDFA restoration error was –0.35° ± 1.88°, whereas in Group B it was –0.12° ± 0.94° (*p* < 0.001, Mann–Whitney *U* test). A negative value indicates a more valgus‐oriented femur postoperatively (Table 3).

**Table 3:** This table summarises the radiographic outcomes related to LDFA restoration.

Outcomes	Group A (*N* = 187)	Group B (*N* = 187)	*p* value
LDFA restoration error (°), mean** ± **SD	–0.35 ± 1.88	–0.12 ± 0.94	< 0.001
Patients within ±0.5° of target LDFA (%), *N* (%)	72 (38.5%)	99 (52.9%)	< 0.001
Patients within ±1° of target LDFA (%), *N* (%)	134 (71.7%)	159 (85.0%)	< 0.001
IQR of LDFA error (°)	–1.0 to +1.1	–0.8 to +0.4	0.027

*Note*: Group B (cartilage removed) showed significantly lower mean error and variability in LDFA restoration compared to Group A. A higher percentage of patients in Group B achieved alignment within both ±0.5° and ±1° of the target.

Abbreviations: %, percentage; °, degree; IQR, interquartile range; LDFA, lateral distal femoral angle; N, number of evaluation cases; SD, standard deviation.

**Figure 4:**
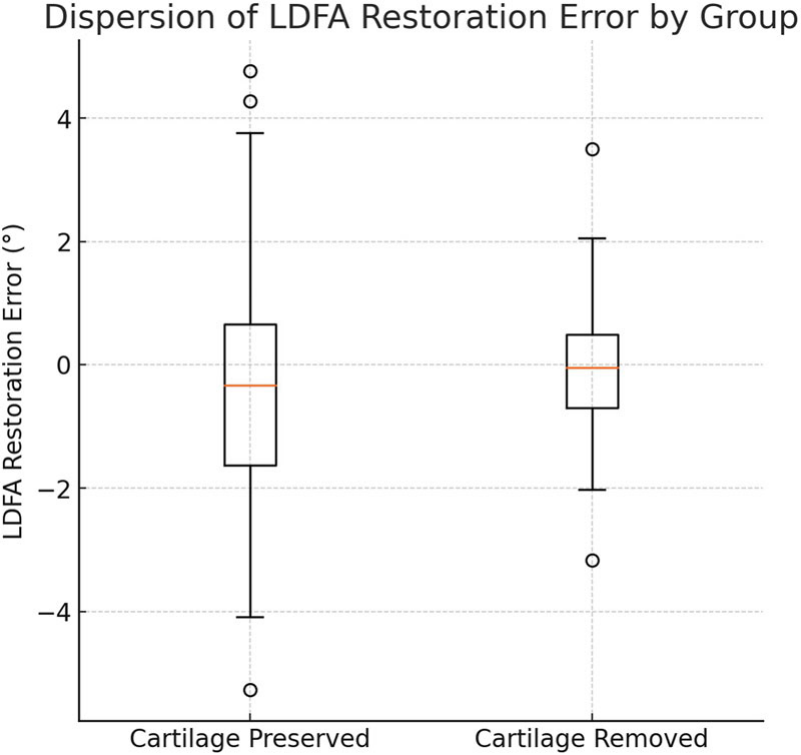
Boxplot illustrating the distribution of LDFA restoration error in the two study groups. The group with cartilage preservation on the unworn femoral condyle (Group A) shows greater variability compared to the group where the cartilage was removed (Group B). The boxes represent the interquartile range (IQR), the horizontal lines within the boxes indicate the median values, and the whiskers extend to the minimum and maximum non‐outlier values. A lower dispersion of LDFA restoration error is observed in the cartilage‐removed group, indicating higher consistency and precision in distal femoral resection. °, degree; LDFA, lateral distal femoral angle.

Levene's test for equality of variances demonstrated a significantly lower dispersion of LDFA restoration error in Group B compared to Group A (*p* = 0.027). Specifically, the interquartile range (IQR) of the LDFA restoration error was 1.0° to 1.1° in Group A and –0.8° to 0.4° in Group B, indicating greater consistency in the group where cartilage was removed (Figure 4).

**Figure 5:**
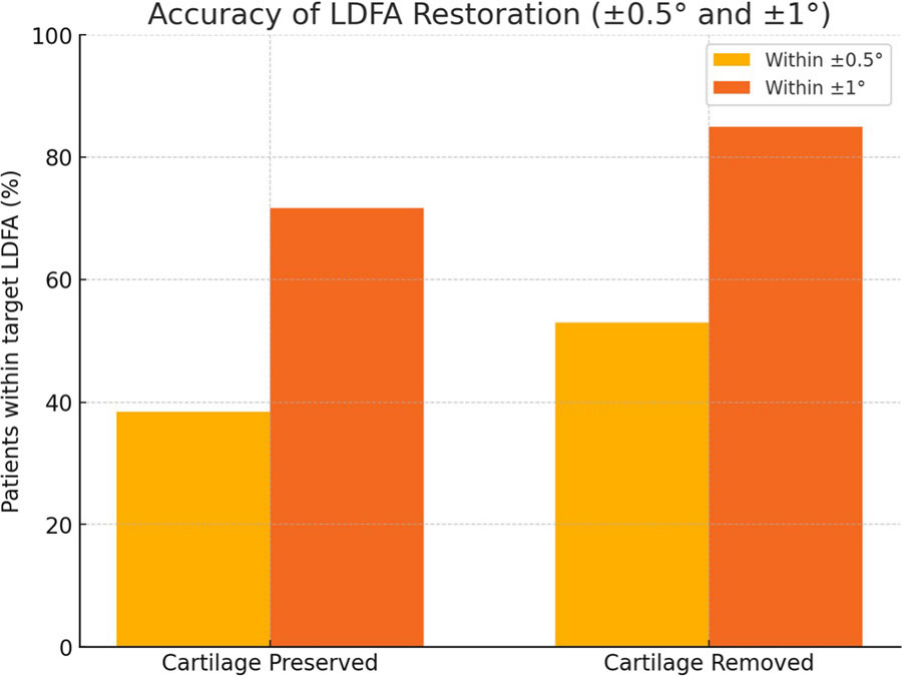
Bar chart showing the percentage of patients in each group who achieved an LDFA restoration error within ±0.5° and ±1° of the preoperative target. A significantly higher proportion of patients in the cartilage‐removed group (Group B) achieved both levels of accuracy compared to the cartilage‐preserved group (Group A), confirming the positive effect of removing cartilage from the unworn femoral condyle on the precision of distal joint line restoration. °, degree; LDFA, lateral distal femoral angle.

The proportion of patients achieving an LDFA restoration error within the following ranges was significantly higher in Group B: Within ±0.5° of target LDFA: 52.9% in Group B versus 38.5% in Group A (*p* < 0.001); Within ±1° of target LDFA: 85.0% in Group B versus 71.7% in Group A (*p* < 0.001) (Figure 5).

## DISCUSSION

The most important finding of this study is that removing cartilage from the unworn femoral condyle significantly improves the accuracy and consistency of distal femoral joint line restoration in KA‐TKA. Specifically, patients who underwent cartilage removal demonstrated both a lower mean LDFA restoration error and a significantly reduced dispersion of values compared with those who preserved cartilage. Moreover, a higher proportion of patients in the cartilage‐removed group achieved an LDFA restoration within both ±0.5° and ±1° of the target.

Although the mean between‐group difference in LDFA error was less than 1°, the dispersion decreased by approximately 50%, and the proportion of cases within narrow tolerance bands increased substantially. Even minimal angular improvements that consistently maintain resections within ±0.5° to ±1° may enhance reproducibility, reduce coronal outliers, and facilitate tibial planning and soft‐tissue balance in KA workflows. The clinical significance of this radiographic improvement remains to be confirmed through functional outcomes and long‐term follow‐up.

**Figure 6:**
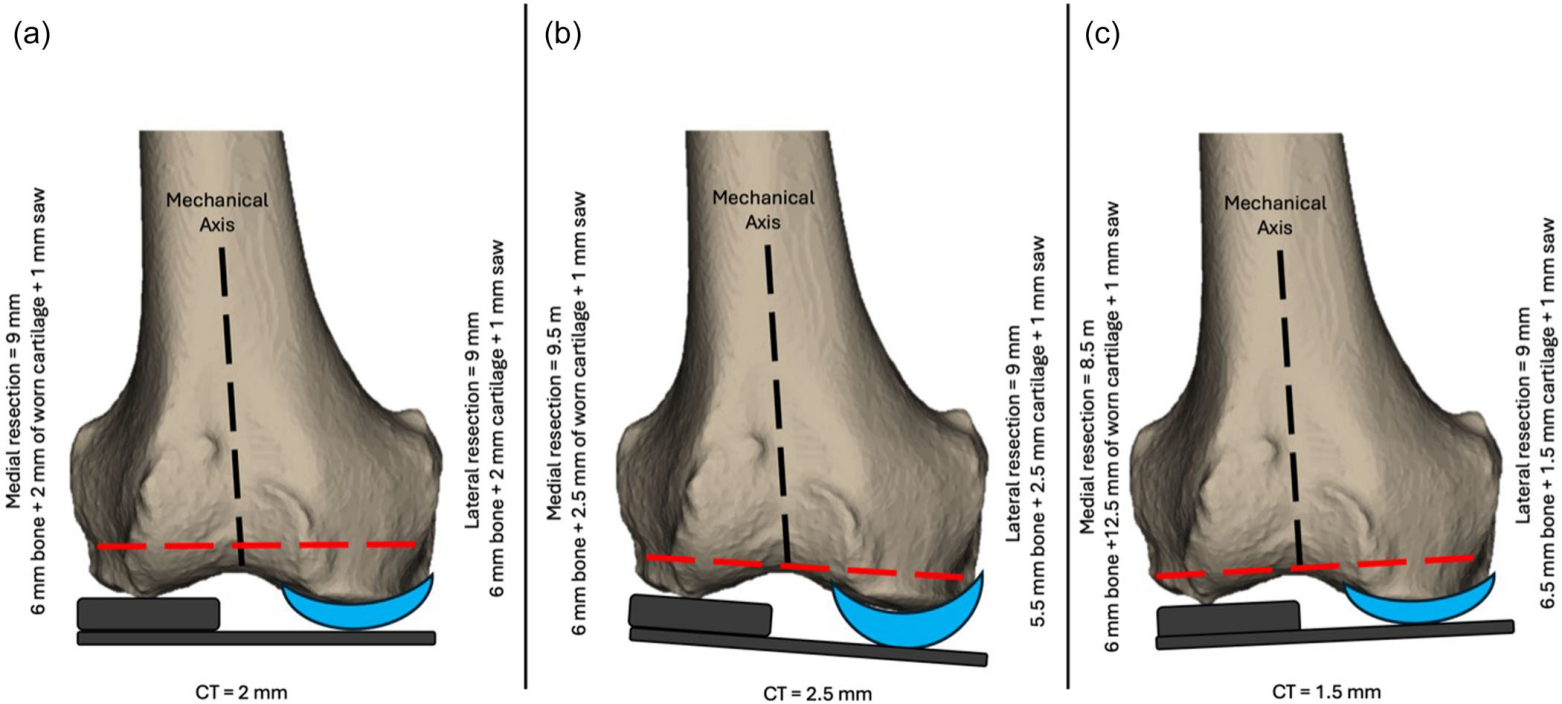
(a) In the standard scenario with 2 mm of cartilage on the unworn condyle, a resection of 6 mm bone + 2 mm cartilage + 1 mm saw thickness results in a total resection of 9 mm on both medial (worn) and lateral (unworn) sides—yielding balanced resections and correct joint line restoration. (b) When actual cartilage thickness is underestimated at 2.5 mm, but the standard 8 mm resection is still performed on the lateral (unworn) side, a dual error occurs: an over‐resection on the medial side (9 mm total) and an under‐resection on the lateral side (8.5 mm total), producing a varus deviation of the joint line. (c) Conversely, suppose cartilage thickness is overestimated at 1.5 mm. In that case, the standard resection causes an under‐resection medially (8.5 mm) and an over‐resection laterally (9.5 mm), leading to a valgus shift of the distal femoral joint line. The red dashed line represents the resulting position of the distal joint line. These scenarios highlight the importance of accurately assessing cartilage thickness to avoid unintended angular malalignment in KA‐TKA. With the technique described in this study, such joint line height errors can be corrected intraoperatively by increasing or decreasing the thickness of the distal femoral resection based on the measured cartilage thickness. KA‐TKA, kinematic alignment‐total knee arthroplasty.

The explanation for these results lies in the anatomical variability of femoral cartilage thickness, particularly on the unworn side. Recent studies have consistently demonstrated that the assumption of a uniform 2 mm cartilage thickness is often inaccurate, with considerable inter‐individual differences [9, 10]. If this variability is not accounted for intraoperatively, the precision of distal femoral resections may be compromised, leading to suboptimal restoration of the distal joint line. By systematically removing the cartilage from the unworn condyle, surgeons eliminate this potential source of error, allowing for a more accurate, reproducible, and reliable bone resection. To better understand the clinical relevance of these findings, it is helpful to consider practical examples related to distal femoral resections during KA‐TKA (Figure 6). Let us assume a femoral component with a distal thickness of 9 mm. To preserve the original joint line level, an equivalent amount of cartilage and bone should be removed, accounting for approximately 1 mm of saw blade thickness. In the first scenario, where the cartilage thickness on the unworn condyle is 2 mm, preserving the cartilage and performing a 6 mm resection on the worn side and an 8 mm resection on the unworn side results in accurate, balanced resections that maintain correct distal femoral alignment. However, if the cartilage thickness on the unworn condyle is underestimated and measures 2.5 mm, applying the same resection protocol (6 mm on the worn side, 8 mm on the unworn side) introduces a dual error: an over‐resection of 0.5 mm on the medial (worn) side and an under‐resection of 0.5 mm on the lateral (unworn) side. This asymmetry produces a varus malalignment of the distal femoral joint line. Conversely, suppose the actual cartilage thickness is only 1.5 mm, and the same resections are performed. In that case, the result is an under‐resection of 0.5 mm on the medial side and an over‐resection of 0.5 mm on the lateral side, leading to a valgus malalignment of the distal femoral joint line.

Although this study specifically focused on the distal femoral joint line and the influence of cartilage thickness on LDFA restoration, similar biomechanical considerations can be reasonably extended to the posterior femoral condyles [9, 10]. The same variability in cartilage thickness that affects distal resections may also be present on the posterior aspects of the femur, potentially introducing inaccuracies in posterior resections if not properly addressed during surgery. Such inaccuracies may lead to unintended alterations in the rotational alignment of the femoral component. This parameter is critically associated with patellar tracking, flexion gap balance, and overall knee kinematics [8]. Although rotational alignment was not assessed in the present study, investigating this aspect would require postoperative computed tomography (CT) imaging, which remains the gold standard for accurately evaluating femoral component rotation. However, the routine use of postoperative CT scans is limited by the high radiation exposure they entail, making their application questionable in large patient cohorts or in standard clinical practice. These considerations emphasise the importance of developing alternative, low‐radiation or intraoperative tools to evaluate and control for potential rotational errors related to posterior cartilage thickness, thereby optimising both coronal and rotational alignment in KA‐TKA [2, 23].

After complete removal of the cartilage from the unworn condyle, the distal resection can be adjusted according to the measured cartilage thickness using a calibrated pin with 0.5‐mm markings. Modifying the resection only when the measured thickness deviates by more than 0.5 mm from the conventional 2 mm assumption helps prevent overcorrections while maintaining the intended joint line height. This simple intraoperative adjustment may further reduce the risk of coronal malalignment, which is associated with interindividual variability in cartilage thickness.

An additional aspect that deserves attention is the crucial role of performing accurate femoral resurfacing as the foundation for achieving proper tibial resection in accordance with the principles of KA. KA requires a precise resurfacing of both the distal and posterior femur, followed by a tibial cut—both in the coronal and sagittal planes—based on the re‐established femoral joint line [21]. The goal is to achieve a rectangular extension gap and a trapezoidal flexion gap with a 1–2 mm lateral opening, reflecting the native soft‐tissue behaviour of the knee. If the femoral resurfacing is performed inaccurately, the only way to achieve appropriate soft tissue balance would be to introduce a corresponding error in the tibial cut, which would compromise the anatomical restoration of the joint line [3, 12]. Therefore, minimizing errors in femoral resurfacing and accurately restoring the distal and posterior femoral joint lines theoretically allows for a more anatomical and reliable restoration of the tibial joint line [22]. The findings of this study further reinforce the importance of technical refinements, such as the systematic removal of cartilage from the unworn condyle, to maximise the accuracy of femoral resurfacing and, consequently, ensure a reliable and reproducible tibial resection.

Several limitations of this study should be acknowledged when interpreting the results. First, although the data were prospectively collected, the retrospective analysis inherently introduces the possibility of selection bias and limits the ability to establish causal relationships. Although the study design aimed to minimise this risk through strict inclusion and exclusion criteria, residual bias cannot be entirely ruled out. Second, the radiographic assessment was based exclusively on long‐leg weight‐bearing radiographs, which, despite being a widely accepted and clinically relevant tool for evaluating coronal alignment, do not provide direct information regarding the rotational alignment of the femoral or tibial components, nor do they allow for detailed evaluation of posterior condylar or trochlear anatomy. The lack of postoperative CT scans represents a limitation, particularly considering that rotational malalignment may also influence joint kinematics and functional outcomes. Third, although a robust a priori power analysis was conducted to ensure adequate sample size to detect differences in alignment accuracy, the study was conducted at a single centre, and all surgeries were performed by a limited number of high‐volume, experienced surgeons. While this approach reduces surgical variability and strengthens internal validity, it may limit the external validity and generalisability of the findings to other surgical environments or to surgeons with different levels of experience.

Furthermore, the study focused exclusively on radiographic alignment accuracy as the primary outcome, without incorporating objective functional assessments, gait analysis or Patient‐Reported Outcome Measures. As such, the potential clinical benefits of improved joint line restoration on functional recovery, patient satisfaction, or implant survivorship remain speculative and require dedicated long‐term investigation. Finally, although the study demonstrated a statistically significant improvement in alignment accuracy and consistency following cartilage removal from the unworn condyle, the clinical relevance of a mean difference of 0.23° in LDFA restoration error may be questioned, and further research is necessary to determine whether such radiographic improvements translate into meaningful clinical benefits for patients.

## CONCLUSION

In conclusion, removing the cartilage from the unworn femoral condyle in KA‐TKA significantly enhanced the radiographic precision of distal femoral joint line restoration. Radiographs demonstrated a more accurate replication of the native LDFA in the cartilage‐removal cohort, with greater alignment with the intended target and markedly reduced inter‐patient variability. These findings underscore the importance of accounting for individual differences in cartilage thickness: the traditional “one‐size‐fits‐all” assumption of a uniform ~2 mm cartilage layer is not universally valid. It may compromise the accurate restoration of a patient's unique anatomy. By contrast, a personalised approach to joint line restoration—tailoring bone resections to each patient's actual cartilage wear pattern rather than relying on fixed assumptions—proved more effective in restoring the joint line.

## AUTHOR CONTRIBUTIONS

Giorgio Cacciola conceived and designed the study, analysed the data, and drafted the manuscript. Daniele Vezza contributed to data analysis, literature review, and manuscript editing. Francesco Carturan conducted radiographic measurements and participated in data collection. Francesco Bosco and Alessandro Massè provided critical revision of the manuscript and supervised the project. Luigi Sabatini performed the surgeries, contributed to intraoperative data acquisition, and participated in manuscript review. All authors read and approved the final version of the manuscript.

## CONFLICT OF INTEREST STATEMENT

Luigi Sabatini is a paid consultant by Medacta International. Other authors declare no conflict of interest.

## ETHICS STATEMENT

123/2024 AOU Città della salute e della scienza, Turin, Italy. All patients included in the study provided written informed consent to participate. The consent was obtained at the first postoperative follow‐up visit, after appropriate explanation of the study purpose and procedures.

## Data Availability

The data that support the findings of this study are available on request from the corresponding author. The data are not publicly available due to privacy or ethical restrictions.
